# Effect of mesoporous silica under *Neisseria meningitidis *transformation process: environmental effects under meningococci transformation

**DOI:** 10.1186/1477-3155-9-28

**Published:** 2011-07-25

**Authors:** Luciana M Hollanda, Gisele CG Cury, Rafaella FC Pereira, Gracielle A Ferreira, Andreza Sousa, Edesia MB Sousa, Marcelo Lancellotti

**Affiliations:** 1 Department of Biochemistry, Institute of Biology CP6109, State University of Campinas UNICAMP, CP: 6109-CEP 13083-970, Campinas, SP, Brazil; 2 National Commission of Nuclear Energy, Center of Development of Nuclear Energy, Nanotechnology Services. CP: 941-CEP 30123-970, Belo Horizonte, MG, Brazil

## Abstract

**Background:**

This study aimed the use of mesoporous silica under the naturally transformable *Neisseria meningitidis*, an important pathogen implicated in the genetic horizontal transfer of DNA causing a escape of the principal vaccination measures worldwide by the capsular switching process. This study verified the effects of mesoporous silica under *N. meningitidis *transformation specifically under the capsular replacement.

**Methods:**

we used three different mesoporous silica particles to verify their action in *N. meningitis *transformation frequency.

**Results:**

we verified the increase in the capsular gene replacement of this bacterium with the three mesoporous silica nanoparticles.

**Conclusion:**

the mesouporous silica particles were capable of increasing the capsule replacement frequency in *N. meningitidis*.

## Findings

Freshly isolated *Neisseria meningitidis *are naturally competent and exchange genetic information with each other by this process. They are also known as a commensal bacterium of the human upper respiratory tract that may occasionally provoke invasive infections such as septicemia and meningitis. This natural competence has been directly correlated to pilliation of these organisms [1] as well as a specific uptake sequence contained multifold within the genome of these bacteria. Pilliated strains are easily transformed by direct incubation with a plasmid containing the uptake sequence or chromosomal DNA [2]. The advantages of doing genetic manipulations within these well-known strains are numerous. Development of systems to construct specific genomic mutations has been used to study their pathogenesis [3-5].

The use of the mutations for the study of the capsular polysaccharide of *N. meningitidi*s allowed the advances in the meningococci pathogenesis understandings [6-8]. The capsular polysaccharide is a major virulence factor and a protective antigen. Meningococcal strains are classified into 12 different serogroups according to their capsular immune specificity, among wich the serogroups A, B, C, Y and W135 are the most frequently found in invasive infections. The capsule of serogroups B, C, Y and W135 strains is composed of either homopolymers (B and C) or heteropolymers (Y and W135) of sialic acid-containing polysaccharides that are specifically linked, depending on the serogroup [9-11]. This polymerization is mediated by the polysialyltransferase, encoded by the *siaD *gene in strains of serogroups B and C (also called *synD *and *synE*, respectively) and by *synG *in serogroup W135. Capsule switching after replacement of *synE*, in a serogroup C strain, by *synG *may result from the conversion of capsule genes by transformation and allelic recombination [3,12,13]. The capsule switching from serogroup C to B *N. meningitidis *was observed in several countries after vaccination campaigns [3,14-17]. It might explain the emergence and the clonal expansion of strains of serogroup W135 of *N. meningitidis *in the year 2000 among Hajj pilgrims who had been vaccinated against meningococci of serogroups A and C [11,18]. These W135 strains belong to the same clonal complex ET-37/ST-11 as prominent serogroup C strains involved in outbreaks worldwide [12,19]. Hence, the emergence of these W135 strains in epidemic conditions raised the question about a possible capsule switching as an escape mechanism to vaccine-induced immunity. Also, these events are expected to occur continuously and can be selected by immune response against a particular capsular polysaccharide [11]. However, the interference of immune response with transformation efficacy has not yet been evaluated. Specific capsular antibodies are expected to bind to the bacterial surface and hence the interference in DNA recognition and uptake.

In addition, environmental interference on the transformation process of this bacterium is also unknown. This work aimed at the use of different mesoporous silica SBA-15, SBA-16 and [SBA-15/P(*N*-iPAAm)], an organic-inorganic hybrids systems based on mesoporous materials and stimuli-responsive polymers, for the study of these nanostructures effect on the transformation process of meningococci, specifically their functions on capsular switching process. Mesoporous silica materials are a fairly new type of material that has pores in the mesoscopic range of 2-50 nm. The characteristic features of ordered mesoporous materials are their monodispersed and adjustable pore size in an inert and biocompatible matrix with an easily modified surface. The intrinsic uniform porous structure of this class of compounds with their large specific surface area and pore volume, associated with surface silanol groups, makes these materials suitable as an adsorbent model for studies involving surface phenomena. The methods used in this work verified the effect of mesoporous silica SBA-15, SBA-16 and [SBA-15/P(*N*-iPAAm)] on the transformation of the serogroup C *N. meningitidis *against two different donor DNA obtained from mutants of this microorganism (M2 and M6).

The characteristics of the strains (*N. meningitidis *and *Escherichia coli*) used in this study are described in Table 1. *N. meningitidis *were grown at 37°C under 5% CO2 on GCB agar medium (Difco) containing the supplements described by Taha *et al*, [20]. When needed, culture media were supplemented with erythromycin at 2 μg/ml and spectomycin at 40 μg/ml. *E. coli *strains used for plasmid preparations were DH5**α**.

**Table 1:** Bacterial Strains used in this work

Strain	Characteristics	Origin (Reference)
**DH5∝**	*Escherichia coli *F-, *end*A1, *hsd*R17 c, *sup*E44, *thi-*1, *gir *A96, *rel*A1	[44]
**pLAN45**	Plasmid containing *ΔNMB0065::ΩaaDA*	This work
**pLAN13**	Plasmid containning the fusion of *synG::ermAM*	This work
**C2135**	*Neisseria meningitidis *serogroup C, BIOMERIEUX	INCQS-FIOCRUZ
**W135_ATCC_**	*Neisseria meningitidis *serogroup W135, ATCC35559	INCQS-FIOCRUZ
**M2**	*N.meningitidis *isogenic mutant ΔNMB0065:: Ω*aaDA*	This work
**M6**	W135_ATCC _transformed with pLAN13 to generate a fusioned strain *synG:ermAM*	This work

The mesoporous silica nanoparticles SBA-15, SBA-16 and [SBA-15/P(*N*-iPAAm)] were characterized by Sousa et al. [21]. Both, SBA-15 and SBA-16 are composed of SiO_2 _but the characteristic features of SBA-15 are the presence of channels arranged in a two-dimensional hexagonal structure and wheat like macroscopic morphology with mean sizes in micrometer scale which consist of many ropelike aggregates. On the other hand, SBA-16 is an example of ordered mesoporous silica with a three dimensional cubic cage structure with three dimensional channel connectivity. Also, in SBA-16 the arrays of the ordered and uniform pores can be observed for which each spherical particle is a single crystal arranged in cubic structure.

**Figure 1:**
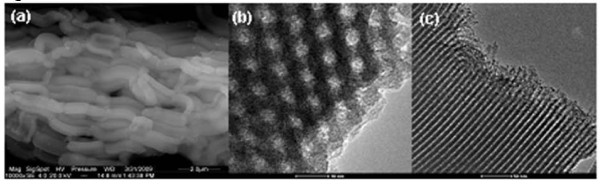
**(a) SEM image of SBA-15 which evidence the presence of elongated, vermicular shaped particles 590 nm wide**. TEM image of SBA-15, which shows a well-defined hexagonal arrangement of uniform pores when **(b)** the incident electron beam was parallel to the main axis of the mesopores and unidirectional channels, and **(c)** the electron beam was perpendicular to the channel axis.

**Figure 2:**
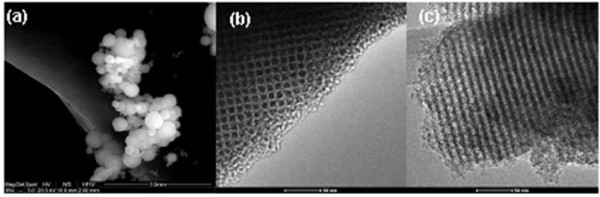
**(a) SEM image of SBA-16 exhibits rounded shape with diameter size between 15 and 20 μm and of an "aggregated morphology"**. TEM images of SBA-16 showed well ordered cubic mesoporous which confirmed the 3D cubic pore structure, when **(b)** viewed along the pore axis and **(c)** perpendicularly to the pore axis.

The SEM images of SBA15 evidence the presence of elongated, 590 nm-wide vermicular shaped particles. SBA-15 consists of many rope-like domains with average sizes of 1.7 μm aggregated into wheat-like macrostructures, Figure 1(a). A similar morphology is observed after the polymerization of P(N-iPAAm) inside the SBA-15 network, presenting 450 nm width (data not showed). TEM image of SBA-15 shows a well-defined hexagonal arrangement of uniform pores when the incident electron beam was parallel to the main axis of the mesoporous (Figure 1b), and unidirectional channels, when the electron beam was perpendicular to the channel axis (Figure 1c). The SBA-16 particle observed from SEM exhibits rounded shape with diameter size between 15 and 20 μm with an "aggregated morphology". The corresponding TEM images of the SBA-16, Figure 2, showed well arranged cubic mesopores what confirmed the 3D cubic pore structure.

Some mesoporous textural properties of SBA-15 and SBA-16 were obtained by the nitrogen adsorption measure. Table 2 summarizes these properties of SBA-15 and SBA-16. BET-specific surface area, S_BET_, was calculated from adsorption data in the relative pressure interval P/P_0 _= 0.045-0.25. A cross-sectional area of 0.162 nm^2 ^was used for the nitrogen molecule in the BET calculations. The total pore volume, V_p_, was calculated from the amount of N_2 _adsorbed at the highest P/P_0 _(P/P_0 _= 0.99). The pore diameter, D_BJH_, was calculated using the adsorption branches of the nitrogen isotherms employing the BJH algorithm. SBA-15, SBA-16 and SBA-15/P(*N*-iPAAm) have small pore diameters from 3.7 to 5.7 nm with very narrow pore size distributions (data not shown). Total pore volumes for SBA-15, SBA-16 and SBA-15/P(*N*-iPAAm) can also be calculated to be 0.96 cm^3^/g, 0.49 cm^3^/g and 0.48 cm^3^/g, respectively. SBA-15 has a higher surface area than the SBA-16 and SBA-15/P(*N*-iPAAm).

**Table 2:** N_2 _adsorption results.

Sample	D_BJH _(nm)	S_BET _(m^2^.g^-1^)	V_p _(cm^3^.g^-1^)
**SBA-16**	3.7	550	0.49

**SBA-15**	5.7	672	0.96

**SBA-15/P(*N*-iPAAm)**	3.7	326	0.48

S_BE_T is the specific area, D_BJH _is the average pore diameter and V_p _is the average pore volume.

**Figure 3:**
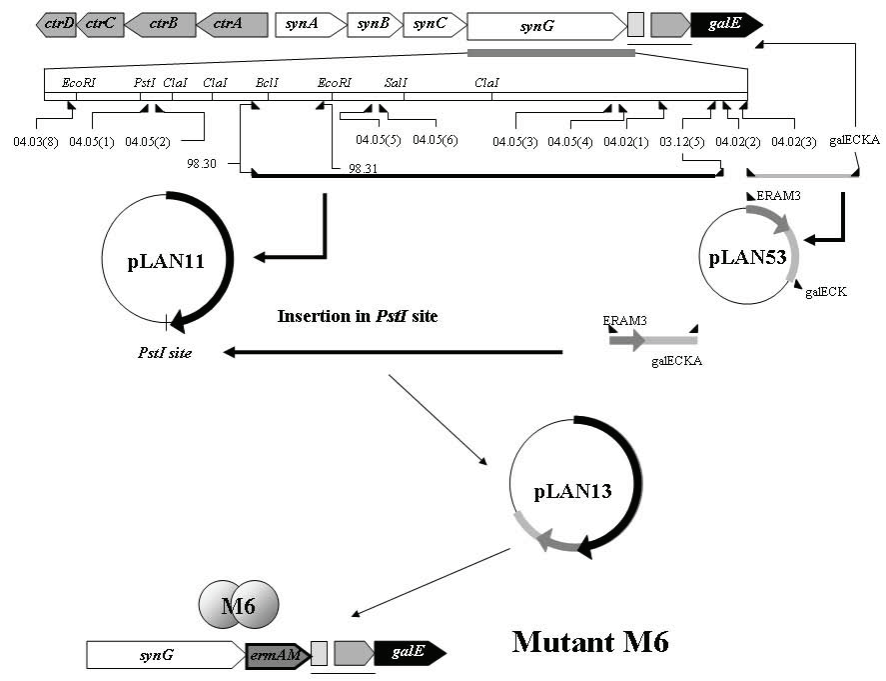
**Schematic representation of the capsule genes of C serogroup in disrupted construction of NMB0065 gene with *aaDA *cassette**. The NMB0065 gene was amplified using the 03-12-3 and 03-12-4 oligonucleotides (Table 3) from C2135 strain. This fragment was cloned into the pGEM-T Easy Vector System II (Promega Corporation, Madison, WI, USA), to generate the plasmid pLAN6. *E. coli *strain Z501 was transformed with plasmid pLAN6 resulting in the plasmid pLAN7. The Ω*aaDA *cassette was inserted into the *BclI *site of pLAN7 to generate plasmid pLAN45, which was transformed into the C2135 strain to generate the isogenic mutant strain M2.

**Figure 4:**
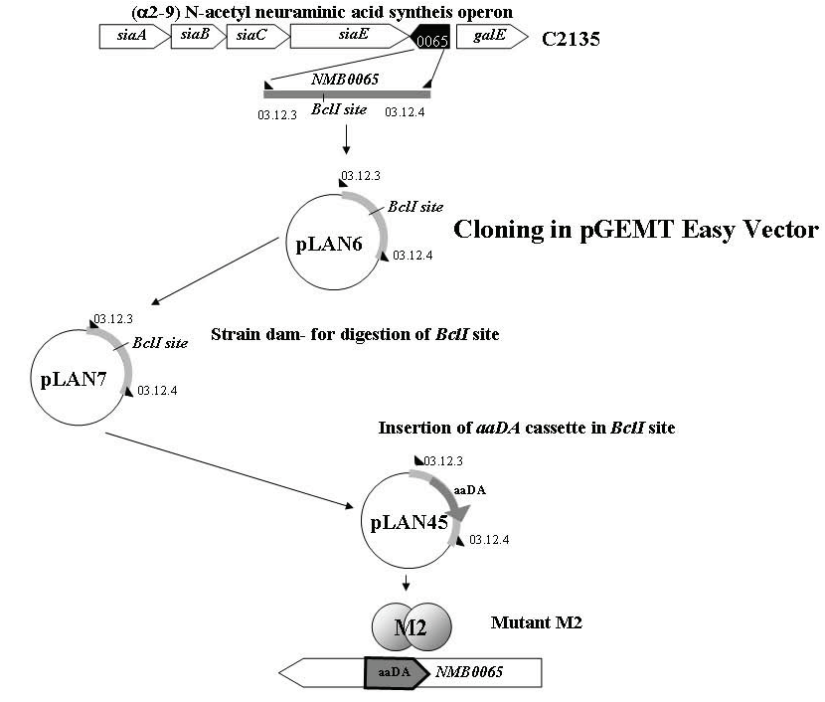
**Schematic representation of the capsule genes of W135 serogroup in transcriptional fusion of *synG *with *ermAM *cassette**. The *synG *gene responsible for the synthesis of the W135 capsule was amplified using the 98-30 and 03-12-5 oligonucleotides (Table 3) from W135_ATCC _strain. The amplified fragment was cloned into the pGEM-T Easy Vector System I (Promega, Madison, WI, USA), to generate the plasmid pLAN11. In the same conditions, another fragment was amplified using the 04.02-2/galECK29A from *synG *downstream sequence to generate pLAN52. The *ermAM *cassette was insered into *Nco*I site of pLAN52 to generate pLAN53. The fragment amplified from pLAN53 with the ERAM1 and galECK29A (Dolan Livengood [23]* et al*., 2003) was insered into *Pst*I site of pLAN11 to generate pLAN13-2. This plasmid was linearised by the enzyme SphI and transformed into W135_ATCC _strain to generate the *synG::ermAM *strain M6, erythromycin resistant.

Recombinant DNA protocols as cloning plasmids, PCR amplifications, insertion of resistance cassettes and transformation were performed as described previously [20,22]. The oligonucleotides used are listed in Table 3. All the mutants obtained by homologous recombination were checked by PCR analysis using a oligonucleotide harboring the target gene and another harboring the cassette. The Figures 3 and 4 describe the design of mutants-M2 and M6, respectively, whose genomic DNA were extracted for gene transfer in C2135 receptor strain.

**Table 3:** Oligonucleotides used in this work

Oligonucleotide	Sequence 5'- 3'	Description
**03.12-3**	TGCGGATCCGCAGTAATTTTATCGGTTGG	NMB0065 forward
**03.12-4**	CCCCACTACCTAAAAAATGCTGATTTG	NMB0065 reverse
**aadA1**	TGCCGTCACGCAACTGGTCCA	Ω*aaDA *forward
**aadA2**	CAACTGATCTGCGCGCGAGGC	Ω*aaDA *reverse
**98.30**	GGTGAATCTTCCGAGCAGGAAA	*synG *forward
**98.31**	AAAGCTGCGCGGAAGAATAGTG	*synG *reverse
03.12-5*	TCGGGATCCTTATTTTTCTTGGCCAAAAA	*synG *reverse
**04.02-1**	CAATGAATCTCGCGTTGCTGTAGGTG	*synG *forward
**04.02-2**	GAAAAATAATTTGGGGCTTAGG	*synG *forward
**galECK29A**	CTTCCATCATTTGTGCAAGGCTGC	*galE *reverse
**ERAM1**	GCAAACTTAAGAGTGTGTTGATAG	*ermAM *forward
**ERAM3**	AAGCTTGCCGTCTGAATGGGACCTCTTTA GCTTCTTGG	*ermAM *reverse

*The underlined sequences in italic are the insertion of the *BamH*I site into original sequence

A preliminary analysis of the action of the mesoporous silica was performed to determine the influence of this nanostructure under *Neisseria meningitidis *growth. The results did not show any influence on bacterial growth of the presence of DNA in addition of SBa15, SBa16 or SBA-15/P(*N*-iPAAm) (data not showed).

The first mutant referent to NMB0065 sequence mutants was the strain M2, this mutant had the NMB0065 sequence from *N. meningitidis *C2135 amplified using 03.12-3 and 03.12-4 oligonucleotides (Table 3). This fragment was cloned into the pGEM-T Easy Vector System II (Promega Corporation, Madison, WI, USA), to generate the plasmid pLAN6. *E. coli *strain Z501 was transformed with plasmid pLAN6 resulting in the plasmid pLAN7. The Ω*aaDA *cassette was inserted into the *BclI *site of pLAN7 to generate plasmid pLAN45, which was transformed into the C2135 strain to generate the strain M2 (Figure 3).

The construction of serogroup W135 mutants with transcriptional fusion *synG*:: *ermAM *was initiated by amplifying the region of *synG *gene using the 98-30 and 03-12-5 oligonucleotides (Table 3) on DNA from the serogroup W135atcc strain. The amplified fragment was cloned into the pGEM-T Easy Vector System I (Promega, Madison, WI, USA), to generate the plasmid pLAN11. Another fragment was amplified using the 04-02-2/galECK29A from *synG *downstream sequence, cloned into pGEM-T Easy Vector, to generate pLAN52. The *ermAM *cassette was amplified by ERAM1/ERAM3 and insered into *Nco*I site of pLAN52 to generate pLAN53. The fragment amplified from pLAN53 with the ERAM1 and galECK29A [23] was inserted into *Pst*I site of pLAN11 to generate pLAN13-2. This plasmid was linearised by the enzyme SphI and transformed into W135_ATCC _strain to generate the *synG::ermAM *fusion strain M6, erythromycin resistant (Figure 4).

**Figure 5:**
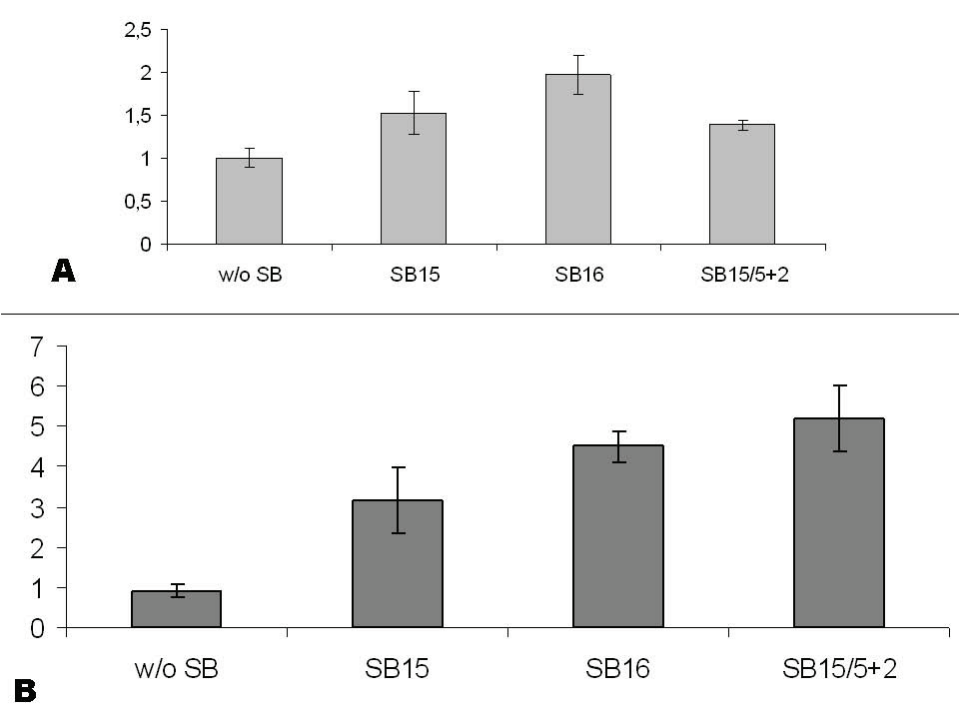
**Graphic of the transformation ratio obtained with In **A**: ratio of transformation of C2135 strain with donor DNA from M2 mutant (ΔNMB0065:: Ω*aaDA)*, In **B**: ratio of transformation of C2135 strain with donor DNA from M6 mutant (*synG:: ermAM*), mimicking a capsular switch replacement, significant analysis of both tests were performed by Tukey test comparing separately each treatment SBA-15, SBA-16 and SBA-15/P(*N*-iPAAm) with the control without nanoparticles (w/o)**.

The analysis of transformation index on SBA-15/SBA-16 nanoparticles action is performed adding of each one in 1.10^8 ^colony forming units (CFU) the receptor strain C2135 was added of 1 μg of M2 or M6 genomic DNA and 30 μg of different mesoporous silica in well plates (table 4 and Figure 5). A negative control was also performed without mesoporous silica. The suspension was incubated at 37°C in CO_2 _atmosphere by three hours in these conditions. The counts of total cfu were performed in GCB spectinomycin or erythromycin plates in triplicate analysis (for M2 and M6 isogenic mutants respectively). The CFU obtained in plates containing specific antibiotic were analyzed by PCR, searching the presence of target gene transfer in the transforming units (Ω*aaDA *cassette for the M2 DNA and *synG *for M6 donor DNA).

**Table 4:** Values obtained from C21 35 Transformation using the donor DNA from M2 and M6 mutants.

Donor DNA (1 μg)	Mean of the UCF transformants obtained in 1.10^8 ^UFC	Ratio (means obtained exposed to silica/mean of negative control)	*P *values (one way Tukey's test)
**Negative Control (without mesoporous silica) M2**	932 ± 175,50	1,00 ± 0,11	
**SBa 15 + DNA M2**	1696 ± 73,30	1,52 ± 0,25	(*P *< 0,05)
**SBa 16 + DNA M2**	1840 ± 423,32	1,97 ± 0,22	(*P *< 0,05)
**SBa 15 (P(*N*-iPAAm) + DNA M2**	1544 ± 358,10	1,38 ± 0,05	(*P *< 0,05)

**Negative Control (without mesoporous silica) M6**	106 ± 10,00	0,92 ± 0,06	
**SBa 15 + DNA M6**	364,33 ± 117,11	3,17 ± 0,80	0,0475 (*P *< 0,05)
**SBa 16 + DNA M6**	558,70 ± 59,56	4,50 ± 0,43	0,0008 (*P *< 0,05)
**SBa 15 (P(*N*-iPAAm) + DNA M6**	598,67 ± 107,56	5,20 ± 0,80	0,0058 (*P *< 0,05)

The graphic of Figure 5 shows significant increase of transformation frequencies using M2 and M6 donor DNA and the mesoporous silica SBA-15, SBA-16 and SBA-15/P(*N*-iPAAm). The use of a different DNA donor had as aim the certification of the independence of mesoporous silica effect on the same bacterial strain-*N. meningitidis *C2135. The analysis of the PCR had demonstrated the transfer of the gene *synG *from M6 donor strains to C2135 receptor strain (data not showed).

The data analyses were made by ratio values between the numbers of transformants CFU obtained with mesoporous silica action by the median value of transformants CFU obtained without silica treatment. The values were analyzed by ANOVA one-way analysis of variance (Tukey test compared each treatment to control without mesoporous silica in transformation). The meningococci growth was not affected by the presence of mesoporous silica (data not shown).

As showed in table 4, the significant values of P < 0.05 obtained in the ratio values between transformation using the donors M2 and M6 mutants DNA, respectively. These values are considered significant when compared with the transformation frequency obtained from negative control without silica action. Thus, the actions of mesopourous silica under the meningococci transformation increased the capacity of the C2135 strains, specially using the construction M6, directly implicated in the capsular switching outbreaks.

Despite the exact mechanism of the capsular switching is still under investigation, we proposed that this process is related to the action of mesoporous silica structures in the transformation frequencies in 1.10^8 ^cfu, with a significant increase when mesoporous silica was used. The behavior of SBA-16, regarding to transformation process of C2135 strain with donor DNA from M2 mutant, was different from that observed for the others. This nanoparticle showed increase of transformation frequency more than SBA-15, and SBA-15/P(*N*-iPAAm) mesoporous silica. Besides the differences in the textural properties showed in Table 2, a probable cause for the different responses is the presence of singular morphological arrangements, as they are hierarchically organized in a special way. Moreover, it is worth noticing that the three-dimensional interconnected pore structure of sample SBA-16 can facilitate the occurrence of adsorption.

The important information is the chromosomal localization of the NMB0065 and *synG *gene. Both are gene of bacterial chromosome and their biological characteristics determined in *Neisseria meningitidis *when these genes are recombined onto chromosomes level. Nevertheless, *N. meningitidis *rarely replicate the plasmids provided from *E.coli *constructions, as those performed in these work (plasmids from pLAN series), exceptionally when in the plasmid carrier antibiotic resistant from another species of *Neisseria *as *N. gonorrhoeae *[24-26].

Also the practical implications of the silica action under meningococci are very important to the workers that usually are exposed at these nanoparticles [27-29]. The careful action of adopting the safety measures not only the silicosis [30-33] but also for adopting safety mesures to prevent not only silicosis but also changing pathology and host adaptation of *N. meningitidis*, will be important in places where silica nanoparticles are present, especially in aerosols. This work is the first to cite the relationships between the silica risks of health caused by meningococcal capsular switching or capsular replacement. This neglected process is described just as an immunologically controlled phenomenon not involving the environmental influences such as the presence of the nanostructures in the atmosphere.

Nevertheless, the capsular switching is described in regions as the sub Saharan Africa [11,34-36] and Saudi Arabia (Hajj pilgrimage) [34,37-43] in desert zones where probably silica nanostructures are present that facilities the capsular switching process. New experiments using the animal models could confirm this hypothesis and has been performed by the research group for *Neisseria meningitdis *and other natural competent bacteria as *Streptococcus pneumoniae *and *Haemophilus influenzae*.

## Competing interests

The authors declare that they have no competing interests.

## Authors' contributions

**LH **carried out the molecular genetic studies; **GC **carried out the Molecular Biology design and plasmids; **RP **carried out the molecular microbiologic tests; **GF **carried out the mesoporous silica electronic microscopy; **AS **carried out the mesoporous silica synthesis; **ES **carried out the mesoporous silica synthesis and design, participated in the sequence alignment and drafted the manuscript; **ML **carried out the molecular genetic studies, participated in the sequence alignment and drafted the manuscript.

All the authors read and approved the final manuscript.
